# Predictive factors for conversion to laparotomy in women undergoing laparoscopic hysterectomy. A re-evaluation of clinicopathological factors in the era of minimally invasive gynaecology

**DOI:** 10.52054/FVVO.16.2.020

**Published:** 2024-06-28

**Authors:** L Lamersdorf, M Tahmasbi Rad, T Karn, B Gasimli, A Bachmann, S Becker, K Gasimli

**Affiliations:** J. W. Goethe-University Frankfurt, Department of Gynecology and Obstetrics, Frankfurt, Germany

**Keywords:** Conversion risk, clinical factors, hysterectomy, laparoscopy, laparotomy

## Abstract

**Background:**

Abdominal hysterectomy has been largely replaced by minimally invasive surgery. Nevertheless, in some situations, a minimally invasive intervention must be converted to laparotomy. Factors associated with conversion to laparotomy are still a matter of debate.

**Objective:**

The aim of this study was to evaluate the clinicopathological factors associated with the conversion of laparoscopic hysterectomy to laparotomy.

**Material and Methods:**

The risk factors for conversion of a preplanned laparoscopic procedure to laparotomy were retrospectively evaluated in 441 patients undergoing a hysterectomy for a benign indication between 2016 and 2020. Associations between the clinical factors were analysed using Pearson’s chi-square and Fisher’s exact test, and predictive values for conversion were assessed through multivariate logistic regression.

**Result:**

Conversion occurred in 32 (7.3%) of the cases. Significant differences were detected for uterus weight (576.9gr vs 174.6gr, p<0.001), myoma size (7.0 cm vs. 1.8 cm, p<0.001), and presence of triple diagnosis consisting of leiomyoma, adenomyosis uteri, and pathological adnexal findings (p<0.013). The conversion resulted in prolonged surgery time (181.6 min vs. 119.6 min, p<0.001) and hospital stay (4.0 vs. 3.1 days, p<0.001), as well as an increased rate of wound infection (15.6% vs. 3.4%, p<0.001). A 10g increase in uterus weight raised the risk of conversion by 7.0%, and a 1cm increase in myoma diameter by 7.3%, while adnexal pathologies and extensive adhesions increased the odds of conversion to laparotomy threefold (ORs of 3.2, 1.09-9.6 and 3.6, 1.3- 10.0, respectively).

**Conclusion:**

Uterus weight, myoma size, the coexistence of pathological adnexal findings, and non-physiological adhesions are independent risk factors for conversion.

**What is new?:**

This study provides data regarding the risk and factors increasing this risk for conversion to laparotomy during laparoscopic hysterectomy.

## Introduction

Hysterectomy is one of the most frequently performed surgical procedures in the field of gynaecology, with an average incidence of 2.1-3.6 per 1000 women per year in Germany (Stang et al., 2011). Abdominal hysterectomy via laparotomy has been gradually replaced by minimally invasive laparoscopic procedures in recent decades. The laparoscopic procedure offers several advantages, such as a shorter duration of surgery, lower rates of intra- and post-operative complications, better post-operative outcomes, and reduced cost to the healthcare system (Warren et al., 2009). Nevertheless, in some situations, a planned laparoscopic intervention must be converted to laparotomy to remove the uterus. Previous studies reported a rate of 4-7% for conversion from laparoscopy to laparotomy with a benign diagnosis (Leonard et al., 2005; Lim et al., 2016; Song et al., 2012; Twijnstra et al., 2013). There are several known clinicopathological factors associated with conversion to and complications from laparotomy in women undergoing laparoscopic hysterectomy. Increased BMI of patients, uterine weight, surgical experience, or adhesive disease due to previous surgeries have been reported as risk factors in several studies (Leonard et al., 2005; Lim et al., 2016; Song et al., 2012; Twijnstra et al., 2013). However, these factors vary widely across studies and their relative importance is still a topic of debate.

The primary objective of this research was to reassess clinical factors contributing to an increased risk of conversion from laparoscopic to abdominal hysterectomy in cases of benign pathology. Additionally, the study aimed to explore the feasibility of conducting pre-operative risk assessments.

## Materials and methods

**Figure 1:**
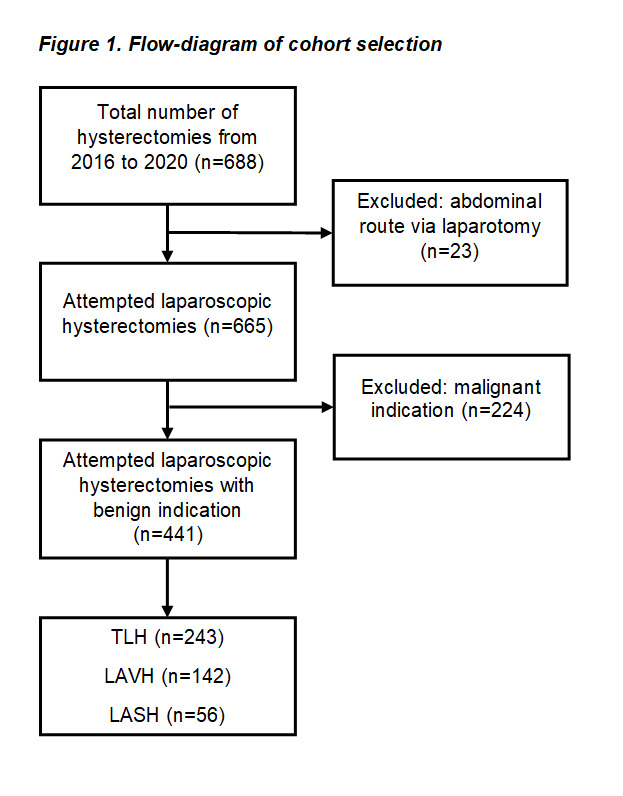
Flowchart of cohort selection.

A total of 441 patients who underwent laparoscopic hysterectomy for benign conditions from January 2016 to December 2020 were enrolled in this analysis. In these cases, either total laparoscopic hysterectomy (TLH) or laparoscopic supracervical hysterectomy (LASH) was performed. The most frequent symptoms of patients leading to surgery were abnormal uterine bleeding disorders and dysmenorrhoea. Cases with inadequate, missing, or insufficient documentation were excluded from our analysis, as well as those with malignant diagnoses. A flow chart of patient selection is presented in Figure 1.

The collected cases were analysed for pre-, intra-, and post-operative characteristics. Patient demographic data and clinically important information, such as BMI, gravidity and parity, previous medical history, and surgeries with a special focus on abdominal or pelvic surgery, were obtained from different medical records. Peri-operative data including indication for procedure, surgical approach, surgical duration, uterine weight, primary surgeon experience level, and complications were taken from corresponding surgery and anaesthesia reports and protocols. Anatomic attachments were all defined as adhesions (beyond those of the rectosigmoidal junction). In addition, the occurrence of attachments in patients with previous surgery, infection or endometriosis were considered to be adhesions. The surgeons were anonymised but classified into experienced and inexperienced. Accurately quantifying surgeon experience is a pervasive challenge in clinical surgical studies, primarily due to the absence of a globally standardised classification system for precise study design. To address this inherent issue and foster transparency, we adopted the certification degree provided by the German Society for Gynecological Endoscopy (Arbeitsgemeinschaft Gynäkologische Endoskopie - AGE e.V.) as a pivotal metric for categorising surgeon experience in our analysis. Surgeons were deemed experienced endoscopic practitioners if they held at least MIC II certification, signifying the highest MIC III level attainable. Notably, the AGE e.V. MIC II certification necessitates that surgeons independently execute a minimum of 400 operative laparoscopies. Post-operative data included length of hospital stay, histologically confirmed diagnoses, and possible subsequent complications, including severe lower abdominal pain, vaginal bleeding, or wound healing disorders. Post-operative histologic diagnoses were stratified as leiomyoma, adenomyosis, cervical intraepithelial neoplasia (CIN), and adnexal pathology (consisting of various cystic findings, adenomas, fibromas, as well as endometriosis) and categorised into single, double or triple diagnoses depending on their co-existence. In addition, we also studied differences that probably resulted from the conversion itself, such as the duration of surgery, wound infection, and prolonged hospital stay. Ultimately, we examined the patient cohort for 32 clinical-pathological factors that may influence conversion from laparoscopy to laparotomy. We considered a “conversion” to be any unplanned intraoperative decision to convert from laparoscopy to laparotomy, whether due to complications or technical reasons.

All surgeries were performed in the Department of Gynecology and Obstetrics at the University Hospital in Frankfurt, Germany.

Postoperative follow-up of all patients was obtained by examination at 6 weeks by their gynaecologist or in our outpatient service. Written informed consent was waived by reason of the retrospective nature of this analysis and patients’ privacy was protected through encoding of personal information. The study was approved by the Institutional Review Boards of the UCT and the Ethical Committee at the University Hospital Frankfurt (project number: UCT-30-2022).

## Statistical analysis

Pearson’s chi-square and Fisher’s exact test were applied to assess associations between categorical parameters. To analyse the predictive value of factors for conversion, univariate logistic regression was used. A stepwise backward multivariate logistic regression model was applied to simultaneously examine the combined effects of multiple factors on conversion. The R software environment (version 3.3.2, http://www.r-project.org/) and SPSS 24 (http://www.ibm.com/) were used for all analyses. All confidence intervals reported are at 95%. All p-values are two-sided and p≤0.05 was considered significant.

## Results

### Patient characteristics

The median age of the patients was 48 years (range: 25-79). The mean BMI was 26 kg/m2 with a range of 15-62 kg/m2. Two hundred fifty-eight (58.5 %) were parous, with 96 (21.8 %) cases having undergone at least one caesarean section. A total of 181 (41.0 %) of the patients had undergone previous abdominal surgery, with 125 (28.1 %) cases having undergone a minimum of one surgery via laparotomy (C-sections included) and 87 (19.7%) patients having undergone at least one laparoscopic surgery for a benign indication. During the study period, laparoscopy was converted to laparotomy in 32 (7.3 %) cases. The baseline characteristics of the patient cohort are shown in Table I.

**Table I:** Patient`s baseline characteristics.

Variables	All patients n=441 (100%)	Converted patients n=32 (7.2%)	Non-converted patients n=409 (92.8%)	p value
Age, years [median]	48 (range: 25-79)	47 (range: 38-66)	48 (range: 25-79)	0.483
BMI, kg/m^2^ [median]	26 (range: 15-62)	27.8 (range: 19-43)	27.3 (range: 15-62)	0.667
Previous abdominal surgeries				
no	260 (59.0%)	19 (59.4%)	241 (58.9%)	0.298
1	105 (23.8%)	5 (15.6%)	100 (24.4%)	
2	44 (10.0%)	6 (18.8%)	38 (9.3%)	
>2	32 (7.3%)	2 (6.3%)	30 (7.3%)	
Previous laparotomy				
no	316 (71.9%)	22 (68.8%)	295 (72.1%)	0.518
1	84 (19.0%)	5 (15.6%)	79 (19.3%)	
>1	41 (9.1%)	5 (15.6%)	35 (8.6%)	
Previous laparoscopy				
no	353 (80.3%)	27 (84.4%)	327 (80.0%)	0.297
1	67 (15.2%)	4 (12.5%)	63 (15.4%)	
>1	20 (4.5%)	1 (3.1%)	19 (4.6%)	
Appendectomy, any types				
no	408 (92.4%)	31 (96.9%)	378 (92.4%)	0.302
yes	32 (7.6%)	1 (3.1%)	31 (7.6%)	
Gravida				
no	183 (41.5%)	19 (59.4%)	164 (40.1%)	0.099
1	77 (17.5%)	2 (6.3%)	75 (18.3%)	
2	91 (20.6%)	7 (21.9%)	84 (20.5%)	
>2	90 (20.4%)	4 (12.5%)	86 (21.0%)	
Spontaneous delivery				
no	257 (58.3%)	25 (78.1%)	232 (56.7%)	0.065
1	66 (15.0%)	3 (9.4%)	63 (15.4%)	
2	67 (15.2%)	4 (12.5%)	63 (15.4%)	
>2	51 (11.6%)	0 (0%)	51 (12.5%)	
Caesarean section				
no	345 (78.2%)	24 (75.0%)	321 (78.5%)	0.708
1	60 (13.6%)	4 (12.5%)	56 (13.7%)	
>1	36 (8.2%)	4 (12.5%)	32 (7.8%)	
Coagulopathy, any types				
yes	14 (3.2%)	1 (3.1%)	13 (3.2%)	1.000
no	427 (96.8%)	31 (96.9%)	396 (96.8%)	
Arterial hypertension				
yes	64 (14.5%)	5 (15.6%)	59 (14.4%)	0.508
no	377 (85.5%)	27 (84.4%)	350 (85.6%)	

Bold values denote statistical significance at the p < 0.05 level ; BMI body mass index.

The most frequent indication for hysterectomy was symptomatic uterine leiomyoma (58.0%), followed by persistent CIN (19.3%) and abnormal uterine bleeding (8.2%). The median operative time was 110 minutes (range: 50-463 min.), and the mean uterine weight was 147 gr (range: 17-1394 gr). Significant adhesions needing adhesiolysis were found in 31.5% of cases. In 98.0 % of the cases, the surgical team was formed by two experienced surgeons or in the presence of at least one MIC II senior surgeon. Intraoperative surgical complications were not observed; however, anaesthetic complications were noted in 16 cases (3.6%), with hypercapnia being the most prevalent issue, occurring in nine cases (2.0%). The primary reason for conversion in our patient cohort was the complexity of operating in a functionally small pelvis and abdomen. Remarkably, none of the laparoscopic hysterectomies during the study period necessitated conversion due to intraoperative complications or access issues.

Table II presents details on surgical indications and procedures performed in the cohort.

**Table II:** Pre-and intraoperative features of study cohort.

Variables			All patients n=441 (100%)	Converted patients n=32 (7.2%)	Non-converted patients n=409 (92.8%)	p value
Indications for hysterectomies						
	Leiomyoma		256 (58.0%)	29 (90.6%)	227 (55.5%)	**0.007**
	CIN		85 (19.3%)	0 (3.1%)	85 (20.8%)	
	Abnormal uterine bleeding		36 (8.2%)	1 (0.0%)	35 (8.6%)	
	Endometriosis		26 (5.9%)	1 (3.1%)	25 (6.1%)	
	Endometrial pathology		14 (3.2%)	0 (0.0%)	14 (3.4%)	
	others		24 (5.4%)	1 (3.1%)	23 (%)	
Operation time, in minutes [median]			110 (range: 50-463)	181.6 (range: 95-463)	119.6 (range: 50-394)	**<0.001**
Uterus weight, in grams [median]			147 (range: 17-1394)	576.9 (range: 101-1394)	174.6 (range: 17-925)	**<0.001**
Skill levels of surgeons* (E=Experienced, U=Unexperienced)						
	EE		220 (49.9%)	18 (56.3%)	202 (49.4%)	0.714
	EU		188 (42.6%)	13 (40.6%)	175 (42.8%)	
	UE		24 (5.4%)	1 (3.1%)	23 (5.6%)	
	UU		9 (2.0%)	0 (0%)	9 (2.2%)	
Adhesions						
	yes		139 (31.5%)	15 (46.9%)	124 (30.3%)	0.073
	no		302 (68.5%)	17 (53.1%)	285 (69.7%)	
Intraoperative complications						
	no		425 (96.3%)	29 (90.6%)	396 (96.8%)	**<0.001**
	yes		16 (3.6%)	3 (9.4)	13 (3.2)	
		Hypercapnia	9 (56.2%)	0 (0.0%)	9 (69.2%)	
		Emphysema	4 (25.0%)	3 (100%)	1 (7.7%)	
		Bradycardia	2(12.5%)	0 (0.0%)	2 (15.4%)	
		Anaphylactic reaction	1 (6.25%)	0 (0.0%)	1 (7.7%)	

Bold values denote statistical significance at the p < 0.05 level; CIN cervical intraepithelial neoplasia; MIC minimal invasive surgery;

*The classification of the surgeons’ experience is based on the MIC classification of the German Society of Gynaecological Endoscopy (AGE) (www.ag-endoskopie.de), which was valid until 2019. Experienced (E) means the surgeons at least with MIC II and III qualification, and unexperienced (U) at least with MIC I.

The mean post-operative hospital stay was three days (range: 1-11). At that time, inpatient observation for three days was standard care following a laparoscopic hysterectomy. As a result of persistent post-operative abdominal pain, return to theatre for a second-look laparoscopy was required in four cases (0.9%) to evaluate for adverse events or complications. However, in all four cases, they revealed no pathological findings on re-laparoscopy. In one case (0.2%) of the whole cohort, there were wound healing disorders as a post-operative complication. In addition, one patient (0.2%) with pre-existing anaemia required the transfusion of one unit of packed red blood cells after a strict evaluation of the indication. Histologically confirmed post-operative diagnoses, either alone or in combination, predominantly included leiomyoma in 329 cases (74.6%) or adenomyosis uteri in 181 cases (41.0%). Histological diagnoses associated with adnexal pathology were identified in 70 cases (15.9%), with the following frequencies: cysts, endometriomas, fibroadenomas, and cystadenomas. Notably, one case required conversion due to the substantial size of the adnexa and uterus, coupled with uncertainty regarding the biology of the adnexal tumour.

Subsequent post-operative histology confirmed the presence of an ovarian fibroadenoma. In 51 cases (12.5%), histological examination revealed a diagnosis of CIN, with 20 cases having a pure CIN diagnosis, distributed as follows: CIN I (2 cases), CIN II (3 cases), and CIN III (15 cases). An additional 16 cases presented with other uterine pathologies, such as fibroids and/or adenomyosis, alongside a CIN diagnosis. A dual diagnosis of leiomyoma and adenomyosis uteri was found in 92 (20.9%) cases and a triple diagnosis of leiomyoma, adenomyosis uteri, and adnexal findings in 21 (4.8%) cases. The mean histologically measured largest diameter of the leiomyoma was 1.9cm (range 1-11cm). Post-operative characteristics of the cases are illustrated in Table III.

**Table III:** Postoperative and histological findings of study cohort.

Variables			All patients n=441 (100%)	Converted patients n=32 (7.2%)	Non-converted patients n=409 (92.8%)	*p* value
Hospital stay, days [median]			3 (range: 1-11)	4.0 (range: 2-11)	3.1 (range: 1-8)	**0.009**
Biggest diameter of leiomyoma, in centimeter [median]			1,9 (range: 0-14)	7.0 (range: 0-14)	1.7 (range: 0-9)	**<0.001**
Postoperative complications						
	no		427 (96.8%)	27 (84.4%)	400 (97.8%)	**<0.001**
	yes		14 (3.2%)	5 (15.6%)	9 (2.2%)	
		Anemia	1 (7.1%)	1 (20.0%)	0 (0.0%)	
		Abdominal pain	12 (85.8%)	3 (60.0%)	9 (100%)	
		Wound healing disorder	1 (7.1%)	1 (20.0%)	0 (0.0%)	
Diagnosis of leiomyoma						
	yes		329 (74.6%)	30 (93.8%)	229 (73.1%)	**0.010**
	no		110 (25.4%)	2 (6.3%)	110 (26.9%)	
Diagnosis of adenomyosis						
	yes		181 (41.0%)	10 (31.3%)	171 (41.8%)	0.268
	no		260 (59.0%)	22 (68.7%)	238 (58.2%)	
Pathological findings of the adnexa						
	yes		70 (15.9%)	8 (25.0%)	62 (15.2%)	0.204
	no		371 (84.1%)	24 (75.0%)	347 (84.8%)	
Double diagnosis						
	yes		92 (20.9%)	4 (12.5%)	88 (21.5%)	0.267
	no		349 (79.1%)	28 (87.5%)	321 (78.5%)	
Triple diagnosis						
	yes		21 (4.8%)	5 (15.6%)	16 (3.9%)	**0.013**
	no		420 (95.2%)	27 (84.4%)	393 (96.1%)	

Bold values denote statistical significance at the p < 0.05 level.

### Factors associated with conversion

In regression analysis, no significant difference in age and BMI between non-converted and converted cases were exposed in our study cohort (p=0.31 and p=0.67, respectively). Similarly, previous abdominal surgery, a caesarean section, or spontaneous delivery, as well as the presence of co-morbidities diseases (e.g., Coagulopathy), demonstrated no significant association with conversion. In addition, we did not detect any influence of the surgeon team (both experienced vs. one experienced and one inexperienced surgeon) on decision-making in favour of conversion. In contrast, a significant difference was observed regarding the uterine weight. The mean weight of the uterus was 576.9 gr (range: 101-1394 gr) for converted and 174.6 gr (range: 17-925 gr) for non-converted hysterectomies (p<0.001). Furthermore, the histologically-measured largest diameter of the leiomyoma had a significant influence on conversion (mean 7.0 cm vs. 1.8 cm, p<0.001, respectively). The presence of adhesions did contribute to an extended duration of surgery and exhibited a trend associated with conversion. However, this trend did not achieve statistical significance (46.9% in converted cases and 30.3% in non-converted cases, p=0.073, respectively). The necessity for conversion due to adhesions occurred in only four cases, a determination based on the experience of the surgical team.

A longer duration of surgery, which increased by 64.5% to a mean of 181.6 minutes (range: 95-463 min.) (p<0.001), a longer hospital stays (4.0 vs. 3.1 days, p<0.001, respectively) and an increase in post-operative complications (15.6% vs. 3.4%, p<0.001, respectively) were observed in the converted cohort. Wound healing disorders as well as anaemia requiring transfusion were seen in one patient (3.1%) in the converted group. None of these complications occurred among non-converted patients (p=0.07). Three (9.4%) of the converted patients suffered from severe post-operative lower abdominal pain and required additional analgesia. This was seen in ten (2.2%) of the non-converted patient group (p=0.049).

A post-operative histological dual diagnosis of leiomyoma and adenomyosis uteri was present in 92 (20.9%) of patients, only 4.3% of whom required conversion. Thus, a dual diagnosis has no significant impact on conversion (p=0.27). However, a post-operative triple diagnosis consisting of leiomyoma, adenomyosis uteri, and an adnexal finding (present in 21 patients, 4.8% of the cohort) led to conversion in 15.6% of these cases and thus had a significant impact on potential conversion (p=0.013). Size of leiomyoma with no other associated diagnosis was linked to conversion in 9.1% of cases (p=0.010), whereas the additional presence of adnexal findings (p=0.204), or adenomyosis uteri (p=0.268) did not have a significant influence.

### Predictive model of conversion

**Figure 2:**
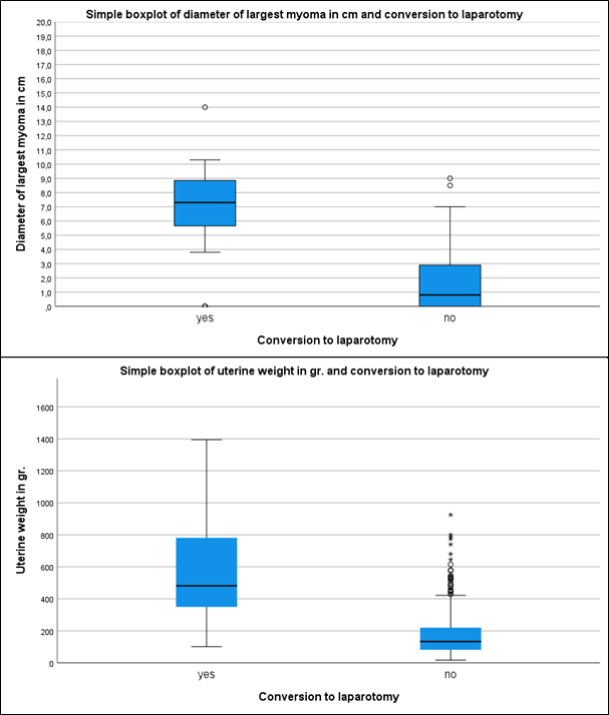
The box plots an illustration of conversion regarding leiomyoma size and uterine weight.

To estimate the influence of multiple factors on the probability of conversion, we applied multivariate stepwise logistic regression. Four variables that contribute to conversion remained significant in the final model as presented in Table IV: uterine weight, any required adhesiolysis at non-physiological sites, a pathologic finding of the adnexa, and the largest measured diameter of the leiomyoma. Based on the odds ratios of these factors, a 10-gram increase in uterine weight increases the probability of conversion by 7.0%. Similarly, a 1-cm increase in the diameter of the largest fibroid increases the probability of conversion by 7.3%. The box plots are illustrated in Figure 2. In addition, the probability of conversion is increased by 3.6-fold when there is an additional pathological finding in the adnexa, and by 3.23-fold when adhesiolysis is required.

**Table IV:** Multivariate logistic regression model for predicting the factors.

Factors		OR for conversion	95% CI	*p*-value
Uterine weight	per gram	1.007	1.00 - 1.01	**<0.001**
Extensive adhesions	yes vs. no	3.6	1.30 - 10.0	**0.014**
Pathological finding of adnexa	yes vs. no	3.23	1.09 - 9.62	**0.034**
Diameter of largest leiomyoma	per cm	1.073	0.94 - 1.22	0.286

Bold values denote statistical significance at the p < 0.05 level; OR Odds ratio; CI confidence interval.

## Discussion

Minimally invasive approaches have become standard care in gynaecological surgery in the last decades and replaced laparotomy for most indications. At present, approximately 70-80% of gynaecological surgeries can be performed by minimally invasive surgery. It is considered the gold standard for some gynaecological diseases such as endometriosis or benign ovarian tumours (Becker and Bokor, 2022; Neis et al., 2016). The conversion rate from laparoscopic to abdominal hysterectomy varies between 4 and 7% in the literature (Leonard et al., 2005; Park et al., 2011; Sokol et al., 2003; Song et al., 2012; Twijnstra et al., 2013). A periodic re-evaluation of risk factors for conversion is interesting in view of the increasing expertise with regards to minimally invasive surgical skills and strategies. In our analysis, uterine weight, extensive adhesions, and coexistence of pathological adnexal findings could established as an independent clinicopathological factor influencing the conversion rate. The higher conversion rate was associated with a prolonged duration of the surgery and a longer hospital stay. These main findings of our current analysis are consistent with the results of most previous studies (Lim et al., 2016; Richards et al., 2021; Sandberg et al., 2016; Twijnstra et al., 2013). Twijnstra et al. (2013) reported in their prospective single- institutional analysis of 1534 patients a significant increase in conversion rates starting at a uterine weight of >500gr (OR, 30.90; p<0.001). In patients of this nationwide Dutch LapTop registration study, BMI and age were also found to be risk factors for conversion. The latter findings are not consistent with our results. However, it is worth remembering that this study is almost 10 years old, and we would assume that during this time, generally speaking, surgical endoscopic skills have improved.

The increased risk for post-operative complications such as wound infections, blood transfusions, or second-look surgery which we saw in the converted group was also reported in other studies (Lim et al., 2016; Park et al., 2011; Richards et al., 2021). Several studies reported the age at surgery, previous abdominal surgery, and the number of caesarean sections as predictive factors for conversion (Sokol et al., 2003; Song et al., 2012; Twijnstra et al., 2013). These factors could not be identified as influencing factors in our analysis. The extensive adhesions were mostly a consequence of prior intra-abdominal surgery, endometriosis, or pelvic infections (Hellebrekers and Kooistra, 2011). Furthermore, the BMI is one of the most debated risk factors for the conversion rate. While several authors reported a significant impact on the conversion rate of BMI above 30m2/kg (Leonard et al., 2005; Lim et al., 2016; Sokol et al., 2003; Song et al., 2012; Twijnstra et al., 2013), it’s clinical relevance could not be confirmed in our analysis. O’Hanlan et al. (2021) recently examined the feasibility and safety of TLH in patients stratified by their BMI. The duration of surgery, estimated blood loss and complication rates of patients with high BMI did not differ significantly from those of patients with normal BMI. The conversion rate was statistically significant, but not generally frequent in their retrospective analysis, most often due to large leiomyoma and hypercapnia. In contrast, an association between hypercapnia and conversion to laparotomy was not demonstrated in our cohort. Therefore, in many cases, the occurrence of anaesthesia-related complications can be prevented pre- and intra-operatively through better coordination between anaesthetists and surgeons. Pre-operative testing and improvement of pulmonary function, as well as using intra-operative low intraperitoneal pressures of 9-12 mmHg, adequate relaxation, and avoidance of emphysema, can reduce the risk of conversion in obese patients.

Since it is known that successful laparoscopic surgery requires a prolonged learning curve, this factor was the subject of several studies. However, (Keurentjes et al., 2018; Sandberg et al., 2016; Twijnstra et al., 2013), the experience and volume of the surgeons could not be confirmed as an independent predictive factor in our study. However, it is difficult to isolate and to assess the surgeon’s role in our study, due to the almost universal participation of at least one senior surgeon in decision-making for conversion (in 98% of cases). Leonard et al. (2005) evaluated the role of sonographic assessment of the leiomyoma and its correlation with the conversion rate. The authors observed that starting from a leiomyoma diameter of 5 cm and larger, leiomyoma diameter became a significant predictive factor for conversion. Our results showed no influence of fibroid size until 7 cm was measured in a histological examination. However, the cut-off value of the largest leiomyoma as a predictive factor remains inconsistent to date in the present literature.

The role of preoperative sonographic evaluation of leiomyoma size in surgical management remains controversial and debated. Moreover, the clinical relevance of different adnexal diseases has not been investigated in coexisting uterine disease in regard to conversion (Sokol et al., 2003). To our knowledge, this is the first study analysing the impact of the coexistence of leiomyoma, adenomyosis uteri, and a pathologic finding of the adnexa on conversion. This could be mostly detected pre-operatively using transvaginal sonography, indicating a prediction for conversion and guiding surgery planning. The pre-operative determination of uterus weight and size in the ultrasonographic examination was the topic of several studies and authors confirmed their accuracy (Dekel et al., 1998; Harb and Adam, 2005; Kung and Chang, 1996; Rovio et al., 2008). Furthermore, some authors assessed the accurate pre-operative determination of pelvic adhesions, and their extent, using ultrasound (Ayachi et al., 2018; Ichikawa et al., 2020). With respect to these results, it seems feasible to determine the most important factors pre-operatively and thus to predict the probability of a conversion to laparotomy. The pre- operative determination of uterine weight, precise measurement of fibroid volume, and identification of extensive and intricate adhesions remained challenging in certain instances. Nonetheless, the assessment and anticipation of uterine volume and adhesions in patients with prior surgeries were feasible through pre-operative imaging modalities (such as ultrasound and clinical examination, or MRI/CT scans). This provided surgeons with the opportunity to engage patients in pre-operative discussions regarding the likelihood of conversion and to meticulously plan the surgical strategy accordingly. Indeed, every case exhibits distinct characteristics, and the attainment of standardisation poses a formidable challenge that necessitates an individualised approach.

Madhvani et al. (2022), in their retrospective analysis of 68,752 women who underwent laparoscopic hysterectomy in benign diseases, developed a predictive model to estimate the risk of conversion to laparotomy. Predictive factors included age, obesity, ethnicity, endometriosis, and adhesions, which would have the strongest predictive value. However, well-evaluated and debated factors such as the exact BMI, size of leiomyoma, and surgical experience were not included in their analysis.

The study’s limitations encompass its retrospective analytical framework and sole reliance on a single institutional database. However, this latter aspect engenders a more cohesive analysis, potentially enhancing the results’ validity through the uniform application of surgical standards by seasoned experts in minimally invasive surgery within the same clinical setting. This precision is further bolstered by the exclusive inclusion of meticulously documented patient cases. Furthermore, due to the retrospective nature of the study, providing an accurate description and classification of the severity of adhesions based on surgical reports posed a challenge. Although our clinic adhered to standard protocols for the classification of adhesions in endometriosis surgeries, the utilisation of this classification or a standardised approach for adhesions in other benign indications was not uniform.

In conclusion, this study undertook a re-evaluation of clinicopathological determinants linked to an elevated conversion rate, culminating in the identification of uterine weight, fibroid dimensions, concurrent pathological adnexal conditions, and the presence of extensive adhesions as independent factors associated with conversion for benign laparoscopic hysterectomy. These anticipated and inferred factors hold the potential to serve as educational instruments during the pre-operative phase, enabling the disclosure of conversion likelihood to patients, and thereby contributing to informed decision-making regarding the optimal surgical approach, whether it be laparotomy or laparoscopy. Moreover, cases situated at the borderline should be subject to meticulous individual assessment and resolution by experienced surgeons.
